# Evaluation of Changes in Beverage Prices and Volume Sold Following the Implementation and Repeal of a Sweetened Beverage Tax in Cook County, Illinois

**DOI:** 10.1001/jamanetworkopen.2020.31083

**Published:** 2020-12-28

**Authors:** Lisa M. Powell, Julien Leider

**Affiliations:** 1 Division of Health Policy and Administration, School of Public Health, University of Illinois Chicago; 2 Institute for Health Research and Policy, University of Illinois Chicago

## Abstract

**Importance:**

Health taxes are policy tools used to reduce harmful consumption of products and raise tax revenue, and they may also be associated with signaling (ie, informational and educational) factors that enhance their impact.

**Objectives:**

To examine changes in prices and volume sold of sweetened beverages following the implementation and repeal of the Cook County, Illinois, Sweetened Beverage Tax (SBT) compared with the comparison site of St Louis County and city, Missouri, which did not impose a tax.

**Design, Setting, and Participants:**

This study used interrupted time series analyses to assess changes in price and volume sold of taxed (based on beverage type and sweetener status) and untaxed beverages in Cook County compared with St Louis following the implementation of the SBT on August 2, 2017, and its repeal effective December 1, 2017. Statistical analysis was performed from January to June 2020.

**Exposures:**

Implementation and repeal of the Cook County SBT.

**Main Outcomes and Measures:**

Changes in taxed and untaxed beverage prices and volume sold. Nielsen food store scanner data were obtained for weekly volume and dollar amount sold of nonalcoholic beverage universal product codes (UPCs) for each site in supermarkets and mass merchandise, grocery, drug, convenience, and dollar stores.

**Results:**

The analytic samples included 16 510 UPCs for volume and 2141 UPCs (balanced sample) for prices for 122 pretax weeks, 16 tax weeks, and 35 postrepeal weeks. Compared with St Louis, posttax implementation in Cook County resulted in a level increase in taxed beverage prices of 1.13 cents per fluid ounce (95% CI, 1.01 to 1.25 cents per fluid ounce), representing a slight overshifting, followed by a posttax repeal level decrease of −1.19 cents per fluid ounce (95% CI, −1.33 to −1.04 cents per fluid ounce), with no resulting change pretax to posttax repeal. Volume sold of taxed beverages in Cook County compared with St Louis exhibited a posttax implementation level decrease of 25.7% (β = −0.297; 95% CI, −0.415 to −0.179) and a posttax repeal level increase of 30.5% (β = 0.266, 95% CI, 0.124 to 0.408), with no net change in volume sold from pretax to 8 months after repeal.

**Conclusions and Relevance:**

This study using interrupted time series analysis found no net change in volume sold of taxed beverages following the implementation and repeal of the Cook County SBT, suggesting the tax had no signaling association. Repeals of such taxes may fully reverse their associations with reduced demand and harms associated with sweetened beverage intake.

## Key Points

**Question:**

Was there a lasting change in beverage prices and volume sold after the 2017 repeal of the Cook County, Illinois, Sweetened Beverage Tax?

**Findings:**

The results from this study using interrupted time series analysis of weekly food store scanner data from the 2 years pretax implementation, the 4 months that the Sweetened Beverage Tax was in place, and the 8 months after repeal found that prices increased and volume sold decreased while the tax was in place, but they returned to their pretax levels following the repeal of the tax.

**Meaning:**

In this study, there was no lasting change in beverage prices or volume sold following the repeal of the Cook County Sweetened Beverage Tax, suggesting that the tax did not change public perception of the harms associated with sweetened beverage intake.

## Teaser

This study using interrupted time series analysis to assess whether the implementation and repeal of a sweetened beverage tax in Cook County, Illinois, were associated with lasting changes in sweetened beverage prices or volume sold.

## Introduction

Goals of health taxes are often 2-fold: (1) reduce consumption of the taxed products deemed to pose health risks and (2) raise tax revenue.^1^ Health taxes have long been imposed on tobacco and alcohol and are increasingly being used as a public health tool to address high levels of sugar-sweetened beverage (SSB) consumption, with more than 40 countries worldwide and local jurisdictions imposing various forms of sweetened beverage taxes.^2^ The health risks associated with SSB consumption, including but not limited to obesity, type 2 diabetes, and cardiovascular disease, have been well documented.^3,4,5,6^

There are 2 key mechanisms through which health taxes may impact consumers’ consumption decisions. First, taxes are expected to be passed on to consumers in the form of higher prices, known as tax pass-through, and, in turn, reduce demand for the taxed products. Second, the fact that the government is implementing a tax on a specific product may signal that its consumption should be reduced.^7^ These signals may be implicit—that is, consumers may infer that because the government is taxing such products they must want consumers to reduce consumption—or signals may be explicit, where information on health harms associated with consumption of the taxed products is provided by governments or stakeholders through media campaigns.

Analyses of explicit media coverage of 2 local-level tax proposals in California revealed that the most common protax messaging provided information on adverse health impacts of SSB consumption, such as type 2 diabetes and obesity, and highlighted opportunities for tax revenue use.^8,9^ Antitax messaging mostly focused on adverse economic impacts and personal choice restrictions; in turn, protax media campaigns responded to antitax claims.^8,9^ Broad media campaigns took place in some US jurisdictions; for example, in Seattle, Washington, campaigns provided information on SSB health-related effects, disparities in SSB consumption, opportunities from tax revenue, and counter-industry arguments.^10,11^ However, Philadelphia, Pennsylvania, which taxed both SSBs and artificially sweetened beverages (ASBs), did not use a public health frame but rather focused messaging on revenue use (ie, universal prekindergarten) to garner public support.^12^ In Cook County, Illinois, which also taxed both SSBs and ASBs, the tax ordinance preamble focused on public health, but the pretax implementation media campaign focused more on filling a budget deficit than on health; public health emerged more prominently in campaigns just prior to implementation and during the 4 months that the tax was in place as part of a strategy to ward off pressure from antitax stakeholders.^13^

Implicit or explicit signaling that informs on SSB-related adverse health consequences may have an impact on consumption independent of the price effect of the tax. However, the extent to which SSB tax-related media campaigns that focus on health claims complement or enhance tax impacts has not been widely studied. A study of the 2014 Mexico SSB tax found awareness of the tax itself and self-efficacy were associated with self-reported changes in and levels of consumption following implementation of the tax, but found no associations with SSB-related health beliefs.^14^ Evidence from the sugary beverage tax in Denmark found purchases of taxed beverages equally responsive to the 2012 tax increase and its 2014 repeal.^15^

A tax cut or repeal offers a quasi-experimental opportunity to examine whether reductions in consumption persist even though the price disincentive has dropped or disappeared. If signaling complements the tax and independently induces consumers to reduce their consumption, we would not expect consumption to return to its pretax levels following a tax reduction or repeal. It is important to assess whether prices fully adjust downward, as otherwise there may be ongoing price effects. Studies of the Danish SSB tax found asymmetric impacts on prices: a study of 2 tax hikes vs a tax cut^16^ and one of a tax hike compared with repeal^15^ found overshifting related to the tax increases but smaller price changes associated with the tax reductions.

The repeal of the Cook County, Illinois, Sweetened Beverage Tax (SBT) offers the opportunity to study the extent to which signaling associated with the tax may persist once the tax is repealed. Since 2015, 8 sweetened beverage taxes have been implemented in the US, with 1 having been repealed. The Cook County SBT, a 1.00 cent per fluid ounce tax on the retail sale of SSBs and ASBs, was implemented on August 2, 2017, and subsequently repealed effective December 1, 2017.^17^ This study drew on weekly food store scanner data and used interrupted time series (ITS) analyses to examine the changes in prices and volume sold of sweetened beverages following the implementation and repeal of the Cook County SBT compared with the comparison site of St Louis County and city (hereafter St Louis), Missouri, which did not impose a tax.

## Methods

### Study Data and Sample

This study used Nielsen food store scanner data on the weekly volume and dollar amount sold of all nonalcoholic beverage universal product codes (UPCs) in their sample of stores by UPC, site, and week. The Nielsen store sample included supermarkets and mass merchandise, grocery, drug, convenience, and dollar stores; these data covered more than 90% of food store sales in Cook County, Illinois.^18^ St Louis, Missouri, was used as the comparison site, matched according to similar demographic and socioeconomic characteristics.^19^ This study was approved by the University of Illinois Chicago institutional review board, which also waived informed patient consent because this study did not involve human participants. This study follows theStrengthening the Reporting of Observational Studies in Epidemiology (STROBE) reporting guideline.

Data covered 16 935 beverage UPCs (excluding powdered drink mixes, frozen juices, fountain drink syrups, and energy shots) from 3 time periods: 122 pretax weeks (March 29, 2015, to July 29, 2017), 16 tax weeks (August 6, 2017, to November 25, 2017), and 35 postrepeal weeks (December 3, 2017, to August 4, 2018). Missing data on beverage characteristics (162 UPCs) and the exclusion of a small number of unsweetened tea and coffee, sports, and energy drinks (263 UPCs) left a total of 16 510 UPCs (10 796 taxed; 5714 untaxed) in the analytical sample for volume (99.6% of original volume). Analyses of prices were balanced on UPCs present in every week in both sites, and included 2141 UPCs (1373 taxed; 768 untaxed) (69.9% of original volume).

### Measures

Beverages were classified as taxed or untaxed with taxed beverages further classified as SSBs or ASBs according to beverage type and sweetener status using information obtained from Nielsen, Label Insight, the US Department of Agriculture Food Composition Databases, and internet research.^20,21^ Price in cents per ounce for each UPC was computed as the total dollar amount of sales (excluding the tax) divided by ounces sold within each site and week with the SBT added to the UPC price per fluid ounce measure based on volume for each taxed beverage. Total volume sold and mean price within each site and week were computed across UPCs. Mean price was weighted according to volume sold of each UPC from June 2016 to May 2017 in Cook County, Illinois, and St Louis, Missouri, in addition to 2-mile border areas surrounding both sites.

### Statistical Analysis

ITS analyses^22^ were conducted to assess changes in the outcomes of prices and volume sold of taxed and untaxed beverages in Cook County compared with St Louis after the Cook County SBT was implemented and subsequently repealed. Models for volume used the log volume as the outcome to allow for interpretation of model results in percentage terms. The estimated models (eAppendix in the Supplement) included time variables, month indicators, an indicator for Cook County (intervention site), and indicators of the posttax and postrepeal periods indicating observations after the SBT was implemented (including after repeal) and repealed, respectively. Interaction terms in the price and volume sold models allowed us to estimate the posttax changes and postrepeal changes in intercept and slope in Cook County compared with St Louis.

Models were fit by feasible generalized least squares, allowing for first-order autoregressive autocorrelation within each site with site-specific coefficients, and a heteroskedastic error structure assuming no correlation between sites. For each outcome, there were no differences in baseline slope (ie, pretax trends) between Cook County and St Louis (eTable in the Supplement). A 2-sided significance threshold was set at *P* < .05. Statistical analyses were conducted using Stata version 15.1 (StataCorp) from January to June 2020.

## Results

### Association With Prices

**Figure 1.**
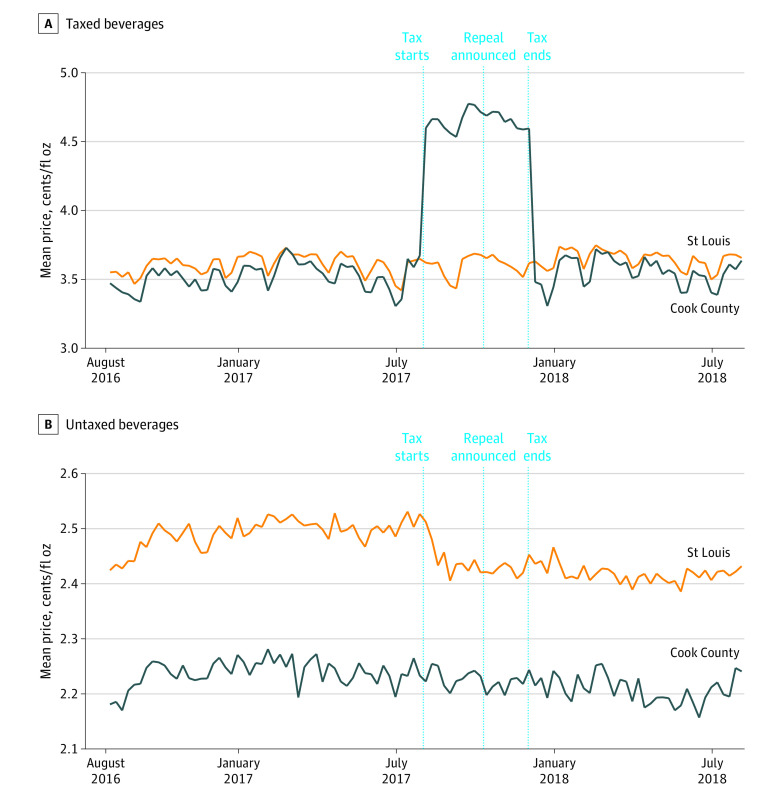
Taxed and Untaxed Beverage Prices in Cook County, Illinois, and St Louis County and City, Missouri, Pretax Implementation, Posttax Implementation, and Posttax Repeal

Table 1 shows that the mean (SD) price of taxed beverages in Cook County during the approximately 2-year pretax implementation period was 3.51 (0.09) cents per fluid ounce. Mean (SD) prices were 4.66 (0.07) cents per fluid ounce during the 4 months that the tax was in place, and then lowered again to 3.55 (0.10) cents per fluid ounce in the 8 months posttax repeal. Mean (SD) prices of taxed beverages in the comparison site of St Louis were generally unchanged from the pretax through posttax repeal period at 3.60 (0.07) to 3.65 (0.07) cents per fluid ounce. Mean prices of taxed SSBs and ASBs followed similar patterns as taxed beverages overall. Mean prices of untaxed beverages in both Cook County and St Louis were generally unchanged. Figure 1 shows that mean prices of taxed beverages increased immediately posttax implementation and then postrepeal decreased back to their pretax level, whereas mean prices of untaxed beverages were unchanged.

**Table 1.** Mean Price and Volume Sold of Taxed and Untaxed Beverages in Cook County, Illinois, and St Louis County and City, Missouri^a^

Variable	Cook County			St Louis		
	Pretax period	Tax period	Postrepeal period	Pretax period	Tax period	Postrepeal period
Price, mean (SD), cents/fl oz						
Taxed	3.506 (0.085)	4.661 (0.070)	3.553 (0.102)	3.601 (0.071)	3.599 (0.080)	3.648 (0.065)
SSB	3.465 (0.079)	4.613 (0.067)	3.507 (0.096)	3.573 (0.069)	3.574 (0.074)	3.620 (0.062)
ASB	3.651 (0.111)	4.827 (0.094)	3.714 (0.132)	3.702 (0.087)	3.685 (0.105)	3.746 (0.080)
Untaxed	2.249 (0.038)	2.224 (0.017)	2.208 (0.025)	2.506 (0.040)	2.432 (0.018)	2.418 (0.015)
Volume sold, mean (SD), millions of fl oz						
Taxed	289.421 (35.554)	206.040 (17.582)	272.372 (33.974)	91.439 (7.824)	88.019 (7.033)	84.000 (6.533)
SSB	231.837 (29.825)	171.026 (16.317)	224.399 (30.113)	64.983 (6.116)	62.612 (5.330)	60.668 (5.272)
ASB	57.478 (6.728)	34.896 (1.628)	47.732 (5.545)	26.380 (2.058)	25.287 (1.905)	23.208 (1.995)
Untaxed	396.373 (33.172)	416.441 (27.732)	421.253 (37.136)	90.056 (5.906)	91.265 (4.401)	92.353 (6.392)

Abbreviations: ASB, artificially sweetened beverage; SSB, sugar-sweetened beverage.

^a^ Data are means across the 122 pretax weeks (from March 29, 2015, to July 29, 2017), 16 tax weeks (from August 6, 2017, to November 25, 2017), and 35 postrepeal weeks (from December 3, 2017, to August 4, 2018). Price was computed as the mean price across universal product codes (UPCs) in each site and week, weighted by volume sold by UPC from June 2016 to May 2017 in Cook County, St Louis, and the 2-mile border areas surrounding both sites. Volume sold was computed as the total across UPCs within each site and week.

Table 2 reports results from the ITS regression models that estimated changes in mean prices in Cook County compared with St Louis from pretax to posttax implementation and posttax repeal. Post-tax implementation, there was an immediate mean level price increase of 1.13 cents per fluid ounce (95% CI, 1.01 to 1.25 cents per fluid ounce), representing a slight overshifting of the tax with a tax pass-through rate of 113%. Mean prices remained level throughout the duration of the tax (ie, no change in slope) and then postrepeal mean prices exhibited a level decrease of −1.19 cents per fluid ounce (95% CI, −1.33 to −1.04 cents per fluid ounce), which fully returned prices to their pretax levels. The estimated overall change in prices from pretax implementation to postrepeal was economically and statistically insignificant at 0.013 cents per fluid ounce (95% CI, −0.11 to 0.13 cents per fluid ounce). Prices of taxed SSBs and ASBs similarly exhibited level increases posttax implementation and level decreases posttax repeal with no overall change in prices from pretax implementation to posttax repeal. Untaxed beverage prices slightly increased posttax implementation (0.06 cents per fluid ounce; 95% CI, 0.01 to 0.11 cents per fluid ounce) with no overall change in prices once the tax was repealed.

**Table 2.** Changes in Taxed and Untaxed Beverage Prices in Cook County, Illinois, Relative to St Louis County and City, Missouri^a^

Beverage type	Coefficient estimates (95% CI)				
	Posttax period		Postrepeal period		Overall change
	Change in level	Change in slope	Change in level	Change in slope	
Taxed	1.131 (1.010 to 1.253)	0.002 (−0.011 to 0.015)	−1.185 (−1.328 to −1.042)	−0.001 (−0.015 to 0.013)	0.013 (−0.106 to 0.131)
SSB	1.110 (0.999 to 1.222)	0.004 (−0.008 to 0.016)	−1.190 (−1.321 to −1.059)	−0.003 (−0.016 to 0.010)	0.008 (−0.102 to 0.118)
ASB	1.206 (1.045 to 1.366)	−0.004 (−0.021 to 0.013)	−1.167 (−1.357 to −0.976)	0.006 (−0.012 to 0.024)	0.029 (−0.124 to 0.181)
Untaxed	0.060 (0.007 to 0.114)	−0.000 (−0.006 to 0.006)	−0.014 (−0.074 to 0.045)	0.000 (−0.007 to 0.007)	0.045 (−0.028 to 0.118)

Abbreviations: ASB, artificially sweetened beverage; SSB, sugar-sweetened beverage.

^a^ Estimates are from interrupted time series models for the change in intercept and slope in mean price in Cook County relative to St Louis after the Cook County sweetened beverage tax was imposed and after it was repealed, controlling for month. Posttax changes are relative to the end of the pretax period, and postrepeal changes are relative to the end of the posttax period. The overall change in Cook County compared with St Louis at the end of the postrepeal period relative to the end of the pretax period was computed as the sum of the 2 change-in-intercept terms and the 2 change-in-slope terms multiplied by the number of weeks to which they applied. Models were fit by feasible generalized least squares, allowing for first-order autoregressive autocorrelation within each site with site-specific coefficients, and a heteroskedastic error structure assuming no correlation between sites. The price measure used for this analysis is the mean price in cents per fluid ounce across universal product codes (UPCs) in each site and week, weighted by volume sold by UPC from June 2016 to May 2017 in Cook County, St Louis, and the 2-mile border areas surrounding both sites. The analysis included 122 pretax weeks (from March 29, 2015, to July 29, 2017), 16 tax weeks (from August 6, 2017, to November 25, 2017), and 35 postrepeal weeks (from December 3, 2017, to August 4, 2018).

### Association With Volume Sold

**Figure 2.**
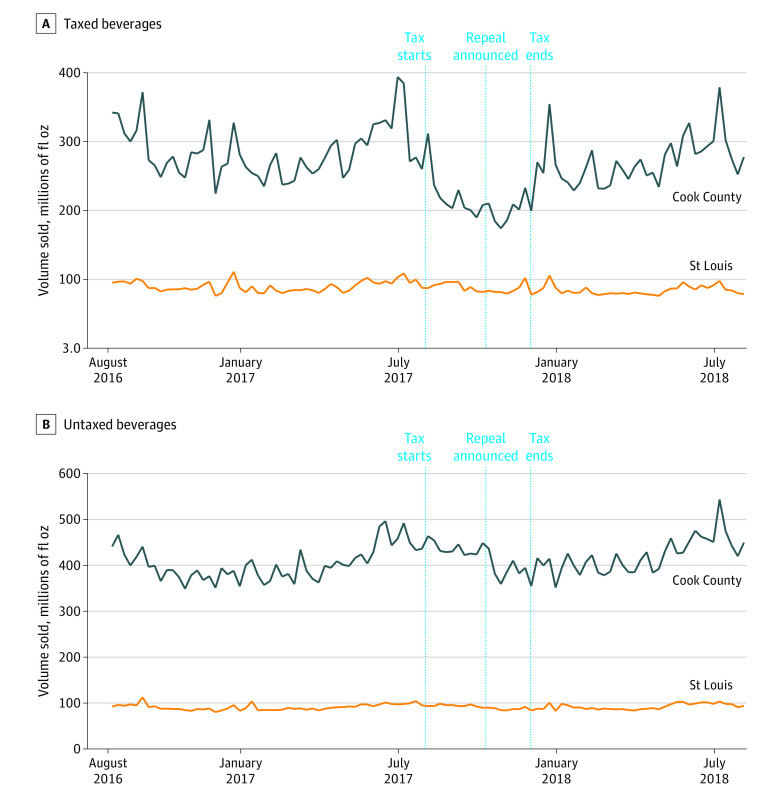
Taxed and Untaxed Beverage Volume Sold in Cook County, Illinois, and St Louis County and City, Missouri, Pretax Implementation, Posttax Implementation, and Posttax Repeal

Table 1 shows that in the approximately 2-year pretax implementation period the mean total weekly volume of taxed products sold in Cook County was 289.42 million fluid ounces, which decreased to 206.04 million fluid ounces per week while the tax was in place and then increased to a weekly mean of 272.37 million fluid ounces during the 8-month posttax repeal period. The volume sold of untaxed products in Cook County, as well as taxed and untaxed products in St Louis, remained generally unchanged (Figure 2).

Table 3 reports the ITS regression estimates of the changes in volume sold in Cook County compared with St Louis posttax implementation and posttax repeal. Volume sold of taxed beverages in Cook County relative to St Louis decreased by 25.7% (β_6_ = −0.297; 95% CI, −0.415 to −0.179) following the implementation of the tax with no significant trend in volume sold over the 4-month period. Subsequently, volume sold increased by 30.5% (β_10_ = 0.266; 95% CI, 0.124 to 0.408) following the repeal of the tax, again with no significant trend over the following 8 months. There was no net overall change in volume sold from pretax to 8-months postrepeal. This pattern held for both taxed SSBs and ASBs. No changes were found in volume sold of untaxed beverages.

**Table 3.** Changes in Taxed and Untaxed Beverage Volume Sold in Cook County, Illinois, Relative to St Louis County and City, Missouri^a^

Beverage type	Posttax period				Postrepeal period				Overall change	
	Change in level		Change in slope		Change in level		Change in slope			
	Coefficient estimates (95% CI)	Exponentiated coefficients	Coefficient estimates (95% CI)	Exponentiated coefficients	Coefficient estimates (95% CI)	Exponentiated coefficients	Coefficient estimates (95% CI)	Exponentiated coefficients	Coefficient estimates (95% CI)	Exponentiated coefficients
Taxed	−0.297 (−0.415 to −0.179)	0.743	−0.002 (−0.014 to 0.011)	0.998	0.266 (0.124 to 0.408)	1.305	0.005 (−0.008 to 0.018)	1.005	0.066 (−0.039 to 0.172)	1.068
SSB	−0.262 (−0.383 to −0.141)	0.770	−0.003 (−0.015 to 0.010)	0.997	0.257 (0.111 to 0.403)	1.293	0.006 (−0.008 to 0.019)	1.006	0.059 (−0.049 to 0.167)	1.061
ASB	−0.461 (−0.581 to −0.341)	0.631	0.005 (−0.008 to 0.018)	1.005	0.328 (0.184 to 0.473)	1.388	−0.003 (−0.016 to 0.010)	0.997	0.013 (−0.093 to 0.120)	1.013
Untaxed	0.036 (−0.034 to 0.106)	1.037	−0.003 (−0.011 to 0.004)	0.997	−0.012 (−0.097 to 0.073)	0.988	0.005 (−0.003 to 0.013)	1.005	0.034 (−0.029 to 0.096)	1.035

Abbreviations: ASB, artificially sweetened beverage; SSB, sugar-sweetened beverage.

^a^ Exponentiated coefficients and overall change were computed to show changes in percentage terms. Estimates are from interrupted time series models for the change in intercept and slope in log volume in Cook County relative to St Louis after the Cook County sweetened beverage tax was imposed and after it was repealed, controlling for month. Posttax changes are relative to the end of the pretax period, and postrepeal changes are relative to the end of the posttax period. The overall change in Cook County relative to St Louis at the end of the postrepeal period relative to the end of the pretax period was computed as the sum of the 2 change-in-intercept terms and the 2 change-in-slope terms multiplied by the number of weeks to which they applied. Models were fit by feasible generalized least squares, allowing for first-order autoregressive autocorrelation within each site with site-specific coefficients, and a heteroskedastic error structure assuming no correlation between sites. The volume sold measure used for this analysis is the total across UPCs within each site and week. The analysis included 122 pretax weeks (from March 29, 2015, to July 29, 2017), 16 tax weeks (from August 6, 2017, to November 25, 2017), and 35 postrepeal weeks (from December 3, 2017, to August 4, 2018).

## Discussion

The estimation results showed that the Cook County SBT was slightly overshifted to consumers in the form of higher prices of taxed beverages, consistent with previous study findings on tax pass-through for Cook County.^19,23^ However, once it was repealed, the outcomes associated with the tax pass-through disappeared with an immediate level price decrease; prices decreased to their pretax levels with no net change in prices of taxed beverages in Cook County compared with changes in St Louis. Pretax to 8-months postrepeal the net overall change in prices of taxed beverages was effectively 0 and statistically insignificant. Thus, if there were to be any persistent reduction in volume sold following the repeal of the Cook County tax, in the absence of persistently higher prices, it could be attributed to a signaling effect. However, the ITS estimation results for volume sold showed that the level decrease in volume sold following the implementation of the tax (consistent with previous study findings on the impact of the tax on volume sold)^18^ was mirrored with a level increase in volume sold once the tax was repealed, with no significant net overall change at 8-months postrepeal.

The absence of evidence of a signaling effect may be explained by the fact that public health was not at the forefront of the Cook County protax campaign messaging leading up to the introduction of the tax.^13^ Furthermore, the explicit health messaging that did occur pretax and during the repeal debate may have been ineffective as it may have seemed defensive, or too little too late. Indeed, an analysis of 374 articles on SSB messaging in the UK in 2014 found that 81% messaged that SSBs have adverse impacts on health and that these messages were consistent across stakeholders. It appears that the intent of these messages was to prime the population on the harms associated with SSBs, as only approximately one-quarter (24%) suggested policy solutions.^24^ Given that the UK SSB tax was not implemented until April 2018, this demonstrates a deliberate public messaging process. There is little published on the effects of media attention during tax adoption or implementation as an independent influence on sales or consumption. A study on the University of California–Berkeley university campus found reductions in sales of taxed beverages during the period following the election outcome (ie, the passing of the SSB tax) but prior to tax implementation in Berkeley, suggesting an independent signaling effect.^25^ In the case of the Cook County SBT, analyses of key informant interviews revealed that protax stakeholders had limited to no time (2 to 3 months) to implement a well-planned campaign.^13^ However, even if the explicit health campaign was limited, the null results for Cook County also indicate that there was no association with implicit signaling from the tax. Additionally, it is worth noting that the tax ordinance required that the shelf price indicate the total price inclusive of the tax amount to be applied at the register but retail outlets were given time to implement this, which may have reduced the salience of the tax during the 4 months that it was in place.

It should be noted, however, that Mexico had a well-planned, lengthy health campaign and, nonetheless, health beliefs were not found to be associated with differences in posttax consumption patterns.^14^ Rather, evaluation results suggested that complementary campaigns providing tools for self-efficacy may offer better opportunities for enhancing the impact of an SSB tax policy,^14^ although the study was cross-sectional and could not determine a causal impact. Also, the study noted that a high proportion of consumers were aware that SSB consumption poses health risks.^14^

Additionally, our results suggesting a null signaling effect are consistent with the recent study^15^ of the 2012 increase in the Danish excise tax on soft drinks (1.08 to 1.58 Danish krones per liter), which was then reduced by half in 2013 and fully repealed in 2014. This tax originated in the 1930s and its primary goal was to generate tax revenue; however, the 2012 increase was part of the 2010 Tax Reform that emphasized tax increases on a number of products harmful to health or the environment (albeit the extent of an explicit SSB-related health campaign is unclear). The study estimated a price elasticity of −1.3 based on both the tax increase and the tax repeal, suggesting that individuals’ price responsiveness was similar for both changes, although the study was limited as it did not have a comparison group.^15^

### Strengths and Limitations

This study has a number of strengths. First, to our knowledge, it is the first study of associations with price and volume sold of a new sweetened beverage tax being implemented (rather than raised) and subsequently repealed and the first ever study of a repeal in the region of the Americas. Second, it uses objectively measured changes in prices and volume sold that covers the vast majority of volume sold in stores. Third, its estimation model includes a comparison site.

Nonetheless, this study is subject to a number of limitations. First, the Cook County SBT was only in place for 4 months, which may not have been enough time to cement behavioral changes related to newly signaled information on health harms associated with SSB consumption. Second, the Cook County SBT did not have a robust explicit public health campaign, and so results may not be generalizable to jurisdictions that provide strong explicit signals on SSB consumption-related health harms. Third, the data do not cover restaurant purchases. Fourth, the data do not allow us to assess differences in volume sold across different household types. With respect to this last point, as shown for price impacts where lower-income populations tend to be more price and tax sensitive,^26,27,28,29,30^ there may also be differences by sociodemographic characteristics in awareness and opinions related to information about SSBs. The Mexico study^14^ on signaling found that higher-income individuals were more aware of the tax compared with lower-income counterparts (74.4% vs 53.1%, respectively) but less likely to believe that the SSB tax was reducing SSB purchases (16.9% vs 26.7%, respectively). Although this study did not assess awareness of public health campaigns, the fact that lower-income individuals were generally less aware of the tax suggests that they may also have been less exposed to the content of the health campaigns related to the SSB tax. A Seattle study of adults’ perceptions of SSB tax-related health and economic impacts found that fewer lower- vs higher-income adults (48% vs 61%, respectively) perceived that the tax would improve public health outcomes.^31^ Thus, future research is warranted to assess that while price impacts may have stronger effects on lower-income households’ purchases, potential signaling effects, if present, may have stronger effects on consumption for higher-income consumers.

## Conclusions

Sweetened beverage taxes are effective policy tools that are associated with higher prices and reduced sales of taxed beverages. We found no evidence of a signaling association from the Cook County SBT, an initiative with a limited public health campaign. Repeals of such taxes may undo their impact on reducing the demand for taxed beverages and, thereby, reverse consumption-related reductions in health harms.

## Notes

Supplement. **eAppendix.** Interrupted Time Series Models of Changes in Prices and Volume Sold **eTable.** Pre-Tax Parallel Trends in Taxed and Untaxed Beverage Prices and Volume Sold in Cook County, Illinois, and St. Louis City/County, Missouri
