# Supplementary material for: Evaluation of Changes in Beverage Prices and Volume Sold Following the Implementation and Repeal of a Sweetened Beverage Tax in Cook County, Illinois
Source: JAMA Netw Open. 2020 Dec 28;3(12):e2031083. doi: 10.1001/jamanetworkopen.2020.31083 (PMC7770557; doi:10.1001/jamanetworkopen.2020.31083)
Supplement: Supplement. — eAppendix. Interrupted Time Series Models of Changes in Prices and Volume Sold eTable. Pre-Tax Parallel Trends in Taxed and Untaxed Beverage Prices and Volume Sold in Cook County, Illinois, and St. Louis City/County, Missouri [file jamanetwopen-e2031083-s001.pdf]

Supplemental Online Content

Powell LM, Leider J. Evaluation of changes in beverage prices and volume sold following the implementation and repeal of a sweetened beverage tax in Cook County, Illinois. *JAMA Netw Open.* 2020;3(12):e2031083. doi:10.1001/jamanetworkopen.2020.31083

eAppendix. Interrupted Time Series Models of Changes In Prices And Volume Sold

eTable. Pre-Tax Parallel Trends in Taxed and Untaxed Beverage Prices and Volume Sold in Cook County, Illinois, and St. Louis City/County, Missouri

This supplemental material has been provided by the authors to give readers additional information about their work.

© 2020 Powell LM et al. *JAMA Network Open.*

eAppendix. Interrupted Time Series Models Of Changes In Prices And Volume Sold

Interrupted time series analyses were conducted to assess changes in price and volume sold of taxed and untaxed beverages in Cook County relative to St. Louis after the Cook County SBT and subsequently after it was repealed. Models for volume used the log volume as the outcome to allow for interpretation of model results in percentage terms. Specifically, models were computed of the form

$$Y = \beta_0 + \beta_1 t + \beta_2 Cook + \beta_3 Cook \cdot t$$
$$+\beta_4 PostTax + \beta_5 PostTax \cdot t + \beta_6 Cook \cdot PostTax + \beta_7 Cook \cdot PostTax \cdot t$$
$$+\beta_8 PostRepeal + \beta_9 PostRepeal \cdot t + \beta_{10} Cook \cdot PostRepeal + \beta_{11} Cook \cdot PostRepeal \cdot t$$

where Y is the outcome (price or log volume), t is time in weeks (numbered from 0-172), $Cook$ indicates Cook County, and $PostTax$ and $PostRepeal$ indicate observations after the Cook County SBT was imposed (including post-repeal) and repealed, respectively. For ease of interpretation, the $PostTax \cdot t$ and $PostRepeal \cdot t$ slope interaction terms were computed to equal 0 for all pre-tax or pre-repeal observations, respectively, and to then be numbered 0, 1, 2, etc. in the post-tax and post-repeal periods, respectively. The $Cook$ interaction terms were computed as usual based on multiplying the $Cook$ indicator by the relevant term. Models also controlled for dummy variables for month, not shown.

The β_0 and β_1 coefficients correspond to the baseline intercept and slope in St. Louis; β_2 and β_3 correspond to the differences in baseline intercept and slope between Cook County and St. Louis; β_4 and β_5 show the change in intercept and slope in St. Louis after the Cook County SBT was imposed; β_6 and β_7 show the post-tax change in intercept and slope in Cook County relative to St. Louis; β_8 and β_9 show the post-repeal change in intercept and slope in St. Louis; and, β_{10} and β_{11} show the post-repeal change in intercept and slope in Cook County relative to St. Louis.

© 2020 Powell LM et al. *JAMA Network Open.*

eTable. Pre-Tax Parallel Trends in Taxed and Untaxed Beverage Prices and Volume Sold in Cook County, Illinois, and St. Louis City/County, Missouri[a]

	Price	Volume Sold
Taxed	0.000<br>(-0.001, 0.001)	0.000<br>(-0.000, 0.001)<br>[1.000]
SSB	0.000<br>(-0.001, 0.001)	0.000<br>(-0.000, 0.001)<br>[1.000]
ASB	0.000<br>(-0.001, 0.001)	-0.000<br>(-0.001, 0.000)<br>[1.000]
Untaxed	0.000<br>(-0.000, 0.000)	0.000<br>(-0.000, 0.001)<br>[1.000]

Abbreviations: ASB, artificially sweetened beverage; SSB, sugar-sweetened beverage.

[a] Coefficient estimates for the pre-tax difference in slopes between Cook County and St. Louis are shown with 95% confidence intervals in parentheses. Exponentiated coefficients giving changes in percentage terms are shown in brackets for volume sold. Estimates are from separate interrupted time series models for the change in intercept and slope in (1) price and (2) volume sold in Cook County relative to St. Louis after the Cook County sweetened beverage tax was imposed and after it was repealed, controlling for month. Models were fit by feasible generalized least squares, allowing for AR(1) autocorrelation within each site with site-specific coefficients, and a heteroskedastic error structure assuming no correlation between sites. The price measure used for this analysis is the mean price in cents per ounce across universal product codes (UPCs) in each site and week, weighted by volume sold by UPC from June 2016 to May 2017 in Cook County, St. Louis, and the 2-mile border areas surrounding both sites. The volume sold measure used for this analysis is the log of the total volume sold across UPCs within each site and week. The analysis included 122 pre-tax weeks (from March 29, 2015 to July 29, 2017), 16 tax weeks (from August 6, 2017 to November 25, 2017), and 35 post-repeal weeks (from December 3, 2017 to August 4, 2018).

© 2020 Powell LM et al. *JAMA Network Open.*
